# Cleavage of Dicer Protein by I7 Protease during Vaccinia Virus Infection

**DOI:** 10.1371/journal.pone.0120390

**Published:** 2015-03-27

**Authors:** Jhih-Si Chen, Hui-Chun Li, Shu-I Lin, Chee-Hing Yang, Wan-Yu Chien, Ciao-Ling Syu, Shih-Yen Lo

**Affiliations:** 1 Department of Laboratory Medicine and Medical Biotechnology, Tzu Chi University, Hualien, Taiwan; 2 Department of Biochemistry, Tzu Chi University, Hualien, Taiwan; 3 Department of Laboratory Medicine, Buddhist Tzu Chi General Hospital, Hualien, Taiwan; George Mason University, UNITED STATES

## Abstract

Dicer is the key component in the miRNA pathway. Degradation of Dicer protein is facilitated during vaccinia virus (VV) infection. A C-terminal cleaved product of Dicer protein was detected in the presence of MG132 during VV infection. Thus, it is possible that Dicer protein is cleaved by a viral protease followed by proteasome degradation of the cleaved product. There is a potential I7 protease cleavage site in the C-terminus of Dicer protein. Indeed, reduction of Dicer protein was detected when Dicer was co-expressed with I7 protease but not with an I7 protease mutant protein lack of the protease activity. Mutation of the potential I7 cleavage site in the C-terminus of Dicer protein resisted its degradation during VV infection. Furthermore, Dicer protein was reduced dramatically by recombinant VV vI7Li after the induction of I7 protease. If VV could facilitate the degradation of Dicer protein, the process of miRNA should be affected by VV infection. Indeed, accumulation of precursor miR122 was detected after VV infection or I7 protease expression. Reduction of miR122 would result in the suppression of HCV sub-genomic RNA replication, and, in turn, the amount of viral proteins. As expected, significant reduction of HCVNS5A protein was detected after VV infection and I7 protease expression. Therefore, our results suggest that VV could cleave Dicer protein through I7 protease to facilitate Dicer degradation, and in turn, suppress the processing of miRNAs. Effect of Dicer protein on VV replication was also studied. Exogenous expression of Dicer protein suppresses VV replication slightly while knockdown of Dicer protein does not affect VV replication significantly.

## Introduction

MicroRNAs (miRNAs) are a group of small noncoding RNAs about 22 nt in length that down-regulate gene expression by either of the two posttranscriptional mechanisms: mRNA cleavage or translational repression [1]. miRNAs have been implicated in a vast array of cellular processes including cell differentiation, proliferation and apoptosis [2]. Links between miRNAs and human diseases such as cancer and neurodegenerative diseases have also been established [3]. Cellular microRNAs could also serve as a barrier against viral infections [4–6].

In the nucleus, primary miRNA transcripts (pri-miRNAs), transcribed by RNA polymerase II, are cleaved by Drosha and DGCR8 [7]. The resulting processed pre-miRNA is recognized by exportin-5 and transited to the cytoplasm [8, 9], where it is then cleaved by Dicer protein [10–13]. The miRNA generated by Dicer is incorporated into the RNA-induced silencing complex (RISC) containing Argonaute proteins (Ago1–Ago4) [2, 14]. Ago-bound miRNA serves as a guide to specifically recognize cellular mRNA so as to either induce their degradation and/or inhibit their translation [1, 2, 14]. Therefore, Dicer is important for the processing of miRNAs.

Cellular RNA interference (RNAi), in addition to microRNAs, could also serve as a barrier against viral infections [4, 14, 15]. RNAi is conserved across the biological species, including invertebrates, plants and animals. Dicer protein is also a key component of RNAi pathway [10–13]. Viruses in turn, have evolved mechanisms that can counteract this anti-viral defense mechanism [4, 15]. Several mammalian viruses expressing different viral proteins with RNA silencing suppressor (RSS) activity have been identified [16–18].

Vaccinia virus (VV), a member of the *Poxviridae* family, is an enveloped DNA virus with a genome of 192 kb encoding about 200 proteins [19]. Various cell lines can be infected by VV, including HeLa, CV-1, mouse L, and chicken CEF cells. VV causes major changes in host cell machinery shortly after infection, and the cytopathic effects (CPE) are observed several hours after infection. The miRNA pathway would involve in the pathogenesis of VV infection, e.g. recent reports demonstrated that VV infection suppresses the cellular microRNA machinery [20, 21]. In addition, VV may possess RSS activity through Dicer protein. The relationships between VV infection and Dicer protein have not been established yet. In this study, we are going to study the effect of VV infection on Dicer protein expression.

## Materials and Methods

### Cell culture

HeLa [22] and HuH7 [23, 24] cells were cultured in Dulbecco's modified Eagle's medium (DMEM) containing 10% fetal bovine serum (FBS), 100 U/ml penicillin and 100 μg/ml streptomycin (Gibco, USA). HCV sub-genomic replicon cells were cultured in DMEM with 10% FBS, 100 U/ml penicillin, 100 μg/ml streptomycin and 400 μg/ml G418 [25]. All cultured cells were maintained at 37°C with 5% CO_2_.

### Plasmid construction and DNA transfection

To clone the DNA fragment for I7L gene coding region, vaccinia genomic DNA [22] was used as template and forward and reverse PCR primers (5’-CGGGGTACCATGGAAAGATATACAGATTTAG-3’ and 5’-ATCGATGGGCCCTTCATCGTCGTTTACTATTC-3’) were used to amplify the gene fragment. After PCR, the DNA fragment was digested by restriction enzymes (*KpnI*/*ApaI*) and cloned into the expression vector pcDNA3.1-V5-His A (linearized by *KpnI*/*ApaI*). To construct I7L gene with mutation in amino acid 328 from Cys to Ala, additional primers (5’-GAAGCCGGGATGTTTATTAGTTTG-3’ and 5’-CCCGGCTTCAGATTCCAACAGCTG-3’) were used following our previous protocols [23, 26].

The plasmid expressing Dicer with T7 tag at the N-terminus was a gift kindly provided by Dr. G.J. Hannon [27]. The site-directed mutagenesis kit used for mutating the potential I7 cleavage site after a.a. 1817 (AG/X) was commercially available (Stratagene, California, USA) and primers (5’-CTTTCTAGAGCCATTTACATGGATAGT -3’ and 5’-GGCTCTAGAAAGCGACTCAAAAATATC -3’) were used to mutate a.a. 1816 and 1817 from AG to SR. The experimental procedures were conducted according to the manufacturer’s instructions.

To clone the plasmid expressing pre-miR122, cDNA library derived from HuH7 cells was used as template and forward and reverse PCR primers (5’GGAATTCGGAGTGTGACAATGGTGTTTG 3’ and 5’GCTCTAGATTTAGTGTGATAATGGCGTTTG 3’) were used to amplify the gene fragment. After PCR, the DNA fragment was digested by restriction enzymes (*EcoRI*/*XbaI*) and cloned into the expression vector pcDNA3 (linearized by *EcoRI*/*XbaI*).

All of the expression plasmids were verified by sequencing.

### Virus infection

Vaccinia virus WR strain [28] was used to infect HeLa and HuH 7 cells in this study, following previously published procedures for virus amplification and plaque assay [22, 29]. Cytosine arabosinide (ara C), where used, was added to the cells at a concentration of 40 μg/ml. Recomninant virus vI7Li, a gift kindly provided by Dr. Bernard Moss, was also used to infect HeLa cells following previously published procedures [30].

Influenza A virus WSN33 was used to infect MDCK cells following previously published procedures for virus amplification and plaque assay [31].

### Western blotting (WB) analysis

Our previous procedures were followed for WB analysis [23, 26, 32]. The primary antibodies used for the analyses in this study were mouse monoclonal antibodies against the C-terminus (a.a. 1813–1912) of Dicer (M01, clone 2F12, Abnova, Taiwan), against the a.a. 378–385 of Dicer (clone 5D12.2, Millipore, USA), against T7 tag (Novagen, USA), against HCV NS5A protein (Biodesign, USA), against V5 tag (Serotec, USA) and rabbit polyclonal antibodies against ERK-2 protein (Santa Cruz Biotechnology, USA), against NPT protein (Upstate, USA), against β -actin (GeneTex, CA, USA) and against I7 protein (a gift kindly provided by Dr. Bernard Moss). Usually, 200 ug protein is enough for the WB analysis. However, due to the insensitivity of the antibodies against the C-terminus of Dicer, 800 ug protein is needed to detect the endogenous Dicer protein. When this antibody was used to detect exogenous T7-Dicer protein from expressing plasmid, the endogenous Dicer protein could not be detected.

### Real-time reverse transcriptase-polymerase chain reaction (real-time RT-PCR)

Total RNAs extracted from HeLa or HuH7 cells (mock or vaccinia virus infection) were converted into cDNAs using oligo-dT as the primer. Our previous procedures were followed for real-time RT-PCR [22, 33]. Specific primers identical to those used in the Takamizawa’s report were used to detect precursor miRNA122 and U6 RNA [34]. Primers (5’ TGGAGACGCCAATAGCAATA 3’ and 5’ TGCTGCTGCAGTGAATTCTT 3’) were used to detect Dicer mRNA while primers (5’CGCTGGTCAGTTCGTGATTA 3’ and 5’AACTCAGGCCCATTTCCTTT 3’) were used to detect TFRC mRNA as a control.

### Statistical Analysis

Experiments were performed three times. Data were analyzed using student t test. P<0.05 was considered statistically significant (p<0.05, *; p<0.01, **; p<0.001, ***).

## Results

### Reduction of Dicer protein during vaccinia virus infection in different cell types

**Fig 1:**
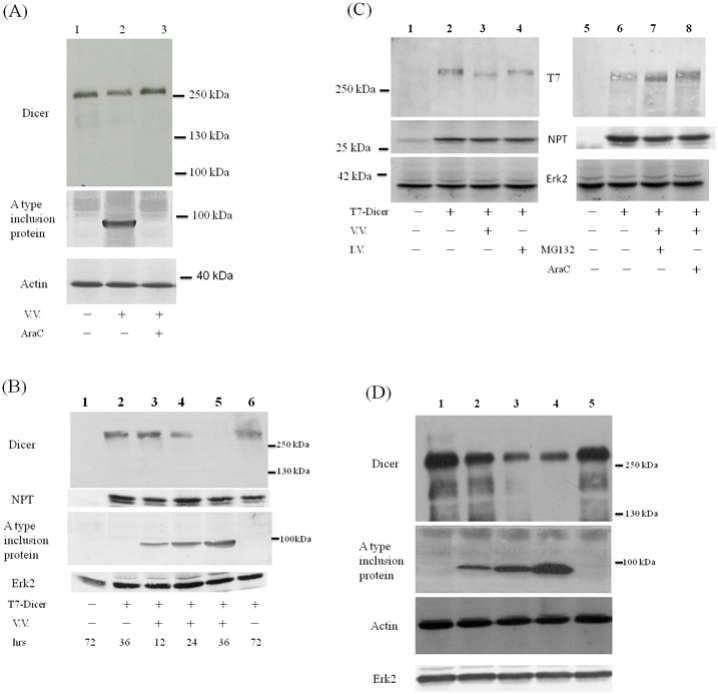
Reduction of Dicer protein during vaccinia virus infection. (A) HeLa cells were either mock infected (lane 1) or infected with VV (M.O.I. = 10) in the absence (lane 2) or in the presence (lane 3) of AraC. Sixteen hrs after infection, cell lysates were analyzed by SDS-PAGE and Western blotting with antibodies against the extreme C-terminus of Dicer protein (upper panel), A type inclusion protein (middle panel) or β -actin protein (lower panel) as the loading control. (B) HeLa cells were either mock transfected (lane 1) or transfected with the plasmid expressing Dicer protein with a T7 tag at its N-terminus (T7-Dicer, lanes 2–6). Forty-eight hrs after transfection, these cells were either mock infected (lane 2) or infected with VV (M.O.I. = 3, lanes 3–5). After the time indicated, cell lysates were analyzed by SDS-PAGE and Western blotting with antibodies against the extreme C-terminus of Dicer protein (upper panel), NPT protein as the transfection control, A type inclusion protein or Erk2 protein (bottom panel) as the loading control. (C) HeLa cells were either mock transfected (lanes 1 and 5) or transfected with the plasmid expressing T7-Dicer (lanes 2–4 and 6–8). Forty-eight hrs after transfection, these cells were either mock infected, infected with VV (M.O.I. = 3, lanes 3, 7 and 8) or with influenza A virus (M.O.I. = 3, lane 4). MG132 (lane 7) or araC (lane 8) was also added in the culture. Twenty-four hrs after infection, cell lysates were analyzed by SDS-PAGE and Western blotting with antibodies against the T7 tag at the N-terminus of Dicer protein (upper panel), against NPT protein (middle panel) as the transfection control, or against Erk2 protein (bottom panel) as the loading control. (D) HuH7 cells were either mock infected (lane 1) or infected with VV in M.O.I = 1 (lane 2), 5 (lane 3) or 10 (lanes 4 and 5). araC was also added in the culture (lane 5). Twenty-four hrs after infection, cell lysates were analyzed by SDS-PAGE and Western blotting with antibodies against the Dicer protein (upper panel), A type inclusion protein (middle panel), β-actin protein or Erk2 protein (lower panel). Both β-actin and Erk2 proteins were served as the loading control.

The amount of endogenous Dicer protein was reduced after VV infection in HeLa cells as detected by antibodies against its extreme C-terminus (lanes 1 and 2, Fig. 1A). This reduction could be recovered after AraC, an inhibitor of VV DNA replication, was added after virus infection (lane 3, Fig. 1A). Unlike the TFRC mRNA decreased after VV infection in a dose dependent manner, the Dicer mRNA level, though decreased, was not specifically reduced after VV infection (Figure SIA in S1 File). Exogenously expressed Dicer protein with a T7 tag at its N-terminus was also reduced after VV but not influenza A virus infection in HeLa cells as detected by antibodies against its extreme C-terminus (Fig. 1B). A smaller protein with tiny amount was detected using antibodies against T7 tag at the N-terminus of this fusion protein (lane 3, left panel of Fig. 1C). This smaller protein was also detected after addition of MG132, an inhibitor of proteasome degradation, after virus infection (lane 7, right panel of Fig. 1C).

The amount of endogenous Dicer protein was also reduced after VV infection in HuH7 cells (Fig. 1D). Again, unlike the TFRC mRNA, the mRNA level of Dicer, though reduced, was not specifically reduced after VV infection in these cells (Figure SIB in S1 File). Similar results were found in HCV replicon cells (data not shown, also see below), which are derived from HuH7 cells with HCV subgenomic RNAs [25].

### Cleavage of Dicer protein by I7 protease facilitates Dicer degradation

**Fig 2:**
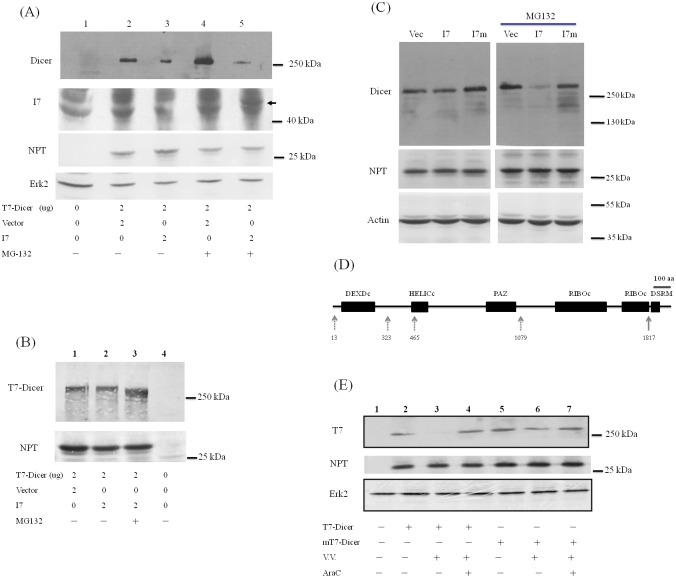
Cleavage of Dicer protein by I7 protease facilitates Dicer degradation. (A) HeLa cells were mock-transfected (lane 1), co-transfected with the plasmids expressing T7-Dicer and empty vector (lanes 2 and 4) or the plasmids expressing T7-Dicer and I7 protease with V5 tag (lanes 3 and 5). Thirty-two hrs after transfection, 10 uM MG132 was also added (lanes 4 and 5). Sixteen hrs later, cell lysates were analyzed by SDS-PAGE and Western blotting with antibodies against the extreme C-terminus of Dicer protein (upper panel), V5-tag to detect the expression of I7 protease, NPT protein as the transfection control, or Erk2 protein as the loading control (bottom panel). The arrow marks the position of I7 in lane 5. (B) HeLa cells were mock-transfected (lane 4), co-transfected with the plasmids expressing T7-Dicer and empty vector (lane 1) or the plasmids expressing T7-Dicer and I7 protease (lanes 2 and 3). Thirty-two hrs after transfection, 10 uM MG132 was also added (lane 3). Sixteen hrs later, cell lysates were analyzed by SDS-PAGE and Western blotting with antibodies against the T7 tag at the N-terminus of Dicer protein (upper panel) or NPT protein as the transfection control. (C) HeLa cells were transfected with empty vector (Vec), plasmids expressing I7 protease (I7) or I7 protease containing C328A mutation (I7m). Twenty-four hrs after transfection, DMSO (left panels) or 20 uM MG132 (right panels) was also added. Twenty-four hrs after treatment, cell lysates were analyzed by SDS-PAGE and Western blotting with antibodies against Dicer (upper panel), NPT protein as transfection control (middle panel) or β-actin for the loading control (bottom panel). (D) Different functional domains in the Dicer protein. Five potential viral I7 protease cleavage sites (a.a. 13, 323, 465, 1079 and 1817) in Dicer protein are marked by arrows. (E) HeLa cells were mock-transfected (lane 1), transfected with the plasmid expressing T7-Dicer (lanes 2–4) or transfected with the plasmid expressing T7-Dicer with mutations in a.a. 1816 and 1817 (mT7-Dicer, lanes 5–7). Twenty-four hrs after transfection, these cells were either mock infected (lanes 2 and 5), or infected with VV (M.O.I. = 5) in the presence (lanes 4 and 7) or absence (lanes 3 and 6) of 100 ug araC. Twenty-four hrs later, cell lysates were analyzed by SDS-PAGE and Western blotting with antibodies against the N-terminal T7 tag of Dicer protein (upper panel), NPT protein as the transfection control or Erk2 protein as the loading control.

It is possible that during VV infection Dicer protein is cleaved by a viral protease at the C-terminus and the cleaved products are then degraded by proteasome degradation pathway. VV G1 protein, a predicted metalloprotease, is essential for the morphogenesis of infectious virions but not for the cleavage of major core proteins [35]. In addition, I7 protease is responsible for processing most or all viral core and membrane proteins in the late stage of VV life cycle [30, 36, 37]. These proteolytic events are involved in the transformation of immature virions into mature virions. There are five predicted cleavage sites for I7 protease (AG/X) in Dicer protein sequence. Therefore, I7 protease may involve in the cleavage of Dicer protein. To address this issue, I7 protease gene was cloned and expressed (Figure SII in S1 File). When co-expressed with I7 protease, exogenously expressed Dicer protein was reduced as detected using antibodies against the extreme C-terminus of Dicer protein (Fig. 2A, lanes 2 and 3). When MG132 was added, full-length Dicer protein was increased in the absence of I7 protease (Fig. 2A, lanes 2 and 4) while it was further reduced in the presence of I7 protease (Fig. 2A, lanes 3–5) as detected using antibodies against the extreme C-terminus of Dicer protein. The same samples were analyzed using antibodies recognizing the N-terminal T7 tag of Dicer protein. A band with smaller size was detected in the presence of MG132 when Dicer and I7 proteins were co-expressed (Fig. 2B, lanes 2 and 3). Expression of I7 protease could only be detected by Western blotting analysis in the presence of MG132 to stabilize this labile protein (lane 5, Fig. 2A). The plasmid expressing a mutant I7 protease lack of the protease activity was constructed by replacing amino acid residue 328 from Cys to Ala [30]. Reduction of Dicer protein was not detected when it was co-expressed with this mutant I7 protein (Fig. 2C).

Five potential viral I7 protease cleavage sites in Dicer protein are after a.a. 13, 323, 465, 1079 and 1817. It is possible that Dicer protein was cleaved by I7 protease after a.a. 1817, and the cleaved protein (about 199 kDa) was further degraded through proteasome ubiquitin degradation pathway (Fig. 2D). To this end, a plasmid expressing the Dicer protein with mutation in a.a. 1816 and 1817 was constructed. Compared with the wild-type Dicer protein, the protein amount of mutated Dicer protein was no longer reduced after VV infection (Fig. 2E).

**Fig 3:**
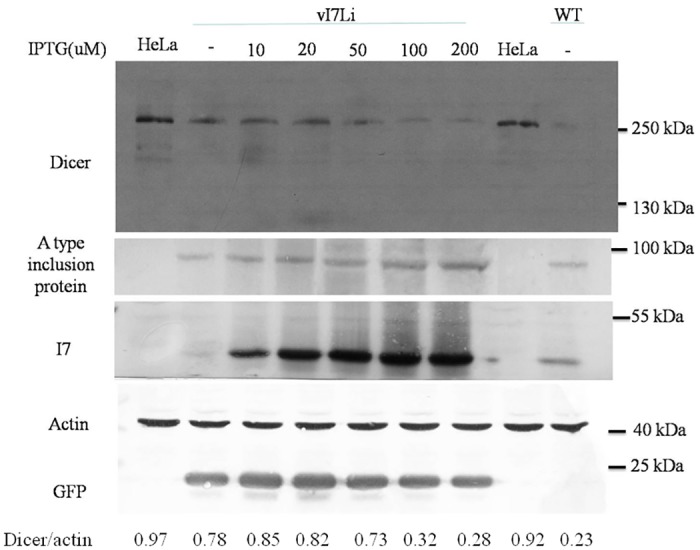
Dicer protein was reduced after the induction of I7 protease expression during recombinant virus vI7Li infection. HeLa cells were mock-infected, infected with wild-type vaccinia virus (WT) or recombinant virus vI7Li (M.O.I. = 5). IPTG was also added as the indicated amount. Twenty-four hrs after infection, cell lysates were analyzed by SDS-PAGE and Western blotting with antibodies against the Dicer, A type inclusion protein, I7, β-actin for the loading control or GFP which is constitutively expressed during vI7Li infection.

To further demonstrate the reduction of Dicer protein by VV infection was through I7 protease, a recombinant VV vI7Li expressing I7 protease protein under IPTG regulation was used [30]. Indeed, Dicer protein was reduced dramatically by recombinant VV vI7Li after but not before the induction of I7 protease (Fig. 3).

### Inhibition of miR122 processing either by vaccinia virus infection or by I7 protease expression

**Fig 4:**
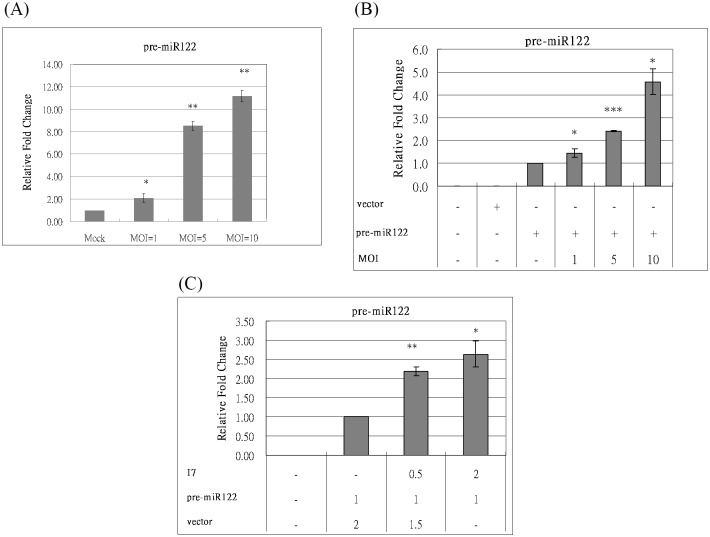
Inhibition of miR122 processing either by VV infection or by I7 protease expression. (A) HuH7 cells were either mock-infected or infected with VV in different M.O.I. (1, 5, and 10). Twenty-four hrs after infection, mRNAs were extracted and converted into cDNA. Then, real-time PCR assay was performed to detect the amount of miR122 using U6 mRNA as the internal control for normalization. (B) HeLa cells were mock-transfected, transfected with empty vector (2 ug) or with the plasmid expressing pre-miR122 (2 ug). Twenty-four hrs after transfection, the cells with pre-miR122 were mock-infected or infected with VV in different M.O.I. (1, 5, and 10). Twenty-four hrs after infection, mRNAs were extracted and converted into cDNA. Then, real-time PCR assay was performed to detect the amount of miR122 using U6 mRNA as the internal control for normalization. (C) HeLa cells were mock-transfected or co-transfected with the plasmids expressing pre-miR122 and I7 protease with the indicated amount. Forty-eight hrs after transfection, mRNAs were extracted and converted into cDNA. Then, real-time PCR assay was performed to detect the amount of miR122 using U6 mRNA as the internal control for normalization. (p<0.05, *; p<0.01, **; p<0.001, ***).

Pre-miRNAs transited by exportin-5 to the cytoplasm were cleaved by Dicer protein to generate miRNAs. Thus, the degradation of Dicer protein should block the formation of miRNAs and results in the accumulation of pre-miRNAs during VV infection. miRNA repertoires are highly cell type specific and change markedly during development or upon cell activation [14]. miR122 is abundant in HuH7 cells [38]. To determine the effect of VV infection on the miRNA processing, the endogenous pre-miR122 level was analyzed in HuH7 cells after VV infection. As expected, the endogenous pre-miR122 level was increased after VV infection in a dose dependent manner (Fig. 4A). Similar results were found in HCV replicon cells after VV infection (data not shown, also see below).

miR122 is scarce in HeLa cells. The plasmid expressing pre-miR122 was constructed and transfected into HeLa cells. The exogenous pre-miR122 level was analyzed in HeLa cells after VV infection. Again, the exogenous pre-miR122 level was increased after VV infection in a dose dependent manner (Fig. 4B) while the Dicer mRNA level was not affected significantly (Figure SIA in S1 File). The exogenous pre-miR122 level was also analyzed in HeLa cells co-transfected with the plasmids expressing pre-miR122 and I7 protease. The exogenous pre-miR122 level was also increased in the presence of I7 protease in a dose dependent manner (Fig. 4C) while the Dicer mRNA level was not affected by I7 protease (Figure SIC in S1 File).

### Inhibition of miR122 function either by vaccinia virus infection or by I7 protease expression

**Fig 5:**
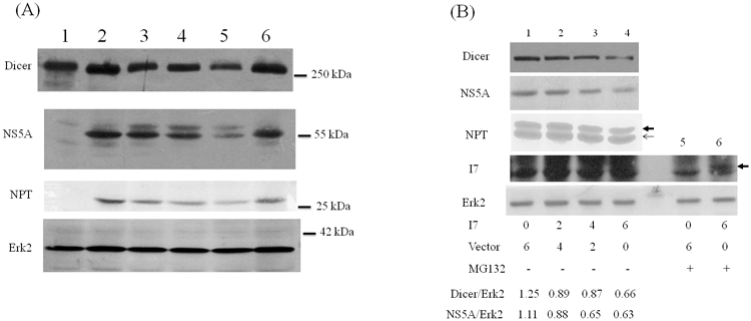
Inhibition of miR122 function either by VV infection or by I7 protease expression. (A) HCV replicon cells were mock-infected (lane 2) or infected with VV in M.O.I. = 1 (lane 3), 5 (lane 4), 10 (lanes 5 and 6). 100 ug araC was also added (lane 6). Twenty-four hrs later, cell lysates were analyzed by SDS-PAGE and Western blotting with antibodies against Dicer protein (upper panel), NS5A and NPT proteins for the expression of HCV subgenomic RNAs, or Erk2 protein as the loading control. Cell lysates from mock-infected HuH7 cells (lane 1) were served as a negative control for the detection of NS5A and NPT. (B) HCV replicon cells were mock-transfected or transfected with the indicated amount of the plasmid expressing I7 protease (lanes 1–6). Thirty-two hrs after transfection, 10 uM MG132 was also added (lanes 5 and 6). Sixteen hrs later, cell lysates were analyzed by SDS-PAGE and Western blotting with antibodies against Dicer protein, NS5A protein for the expression of HCV subgenomic RNAs, NPT protein (the thin arrow marks the position of NPT protein expressed from the transfected plasmids while the thick arrow marks the position of core-NPT fusion protein encoding from HCV subgenomic RNAs.), V5 tag to detect the expression of I7 (The arrow marks the position of I7 in lane 6.) or Erk2 protein as the loading control.

It has been previously reported that miRNA122 could facilitate HCV replication [38, 39]. Degradation of Dicer protein would reduce the production of miRNA122 and, in turn, should repress the HCV RNA replication. Thus, the replication of HCV subgenomic RNA, and in turn the amount of proteins encoded from this RNA, should be reduced in HCV replicon cells after VV infection [33]. Indeed, the HCV NS5A and core-NPT protein levels were suppressed, accompanying with the reduction of Dicer protein in HCV replicon cells after VV infection in a dose dependent manner (Fig. 5A).

The plasmid expressing I7 protease was also transfected into the HCV replicon cells. As expected, accompanying with the reduction of Dicer protein, the HCV NS5A and core-NPT protein levels were suppressed in a dose dependent manner (Fig. 5B). Again, expression of I7 protease could only be detected by Western blotting analysis in the presence of MG132 to stabilize this labile protein (lane 6, Fig. 5B).

### Suppression of VV replication slightly by exogenous expression of Dicer protein

**Fig 6:**
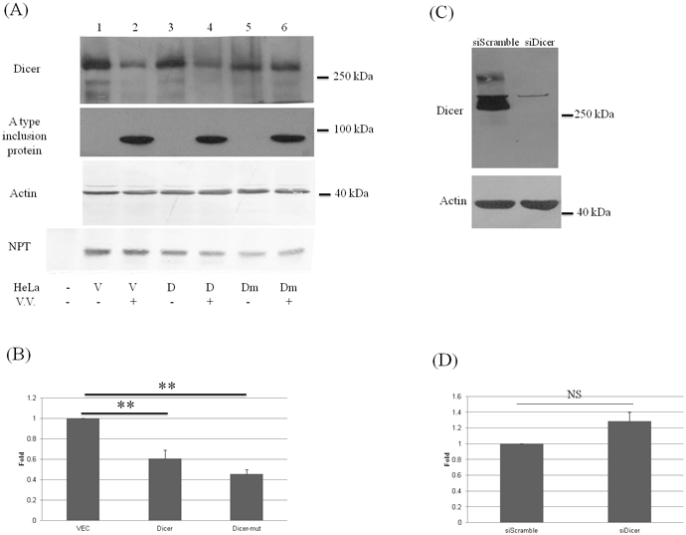
Suppression of vaccinia virus replication slightly by exogenously expressed Dicer protein. (A, B) HeLa cells were transfected with empty vector (2 ug, lanes 1 and 2), with the plasmid expressing T7-Dicer protein (2 ug, lanes 3 and 4) or with the plasmid expressing T7-Dicer with mutations in a.a. 1816 and 1817 (2 ug, lanes 5 and 6). Twenty-four hrs after transfection, the cells were mock-infected (lanes 1, 3 and 5) or infected with VV (M.O.I. = 10) (lanes 2, 4 and 6). Twenty-four hrs after infection, cell lysates were analyzed by SDS-PAGE and Western blotting with antibodies against the Dicer, A type inclusion protein and β-actin (A) while the secreted virus particles from the supernatants (lanes 2, 4 and 6) were analyzed by the plaque assay (B). (p<0.01, **). (C) HeLa cells were transfected with scramble siRNA as a control or siRNA against Dicer. Twenty-four hrs after transfection, cell lysates were analyzed by SDS-PAGE and Western blotting with antibodies against the Dicer (upper panel) and β-actin proteins (bottom panel) as the loading control. (D) HeLa cells were transfected with scramble or Dicer siRNA. Twenty-four hrs after transfection, the cells were infected with VV (M.O.I. = 10). Twenty-four hrs after infection, the secreted viral particles from the supernatants were analyzed by plaque assay. NS: not significant.

To determine the effect of Dicer protein on VV replication, the gain-of-function (over-expression of Dicer) and loss-of-function (knockdown of Dicer) assays were performed. Exogenously expressed Dicer protein suppressed viral protein amount (e.g., A type inclusion protein) inside the cells slightly (Fig. 6A) and also reduced the number of the secreted viral particles in the medium to around 60% (Fig. 6B). The number of secreted viral particles was further reduced when the Dicer protein with mutations in a.a. 1816 and 1817 was exogenously expressed (Figs. 6A and 6B). On the other hand, knockdown of Dicer protein expression using siRNA technology did not increase the number of the secreted VV viral particles significantly (Figs. 6C and 6D).

## Discussion

Our results in this study showed that Dicer protein was reduced in VV-infected cells (Fig. 1), and in turn, the processing and the function of miR122 were blocked (Figs. 4 and 5). Reduction of Dicer protein should affect the processing of universal miRNAs. Indeed, a recent report indicated that Dicer protein was suppressed in VV-infected cells that was associated with a universal reduction of host miRNAs expression [20]. miRNAs have been implicated in a vast array of cellular processes including cell proliferation and apoptosis [2]. Thus, reduction of Dicer protein during VV infection is probably one of the many factors responsible for the viral pathogenesis.

Reduction of Dicer protein in VV-infected cells (Fig. 1) may be caused by several different mechanisms. One possible mechanism is due to mRNA reduction (Figures SIA and SIB in S1 File), which is also reported previously [20]. VV is known to enhance the degradation of host mRNAs by two decapping enzymes encoded by the virus, D9 and D10 [40, 41]. There are still other possible mechanisms. The reduction of Dicer protein during VV infection could be recovered by adding araC to inhibit the VV DNA replication (Fig. 1). Therefore, in addition to the reduction of Dicer mRNA, a viral protease expressed after VV DNA synthesis is also probably responsible for the reduction of Dicer protein. I7 protease is required for AG/X-specific cleavages of viral membrane and core proteins during VV assembly [30]. Our results further demonstrated that Dicer protein was first cleaved by I7 protease after a.a. 1817 and the cleaved product was then degraded by the proteasome-ubiquitin degradation pathway during VV infection (Figs. 1–3). Comparing with the I7 protease encoded by VV infection (Fig. 3), I7 protease derived from expressing plasmid is much more labile because it could only be detected by Western blotting analysis in the presence of MG132 (Figs. 2A and 5B). This also indicated that tiny amount of I7 protease should be sufficient for the cleavage of Dicer protein (Figs. 2A and 5B). However, Dicer protein was reduced after the induction of I7 protease protein in the presence of more than 50 uM IPTG but not in less than 50 uM IPTG (Fig. 3), indicating that not only the tiny amount of I7 protease but also other factors (e.g., whether I7 protease interacts with Dicer protein or not) are important to cleave Dicer protein and facilitate its degradation.

There are many different ways of interactions between viruses and miRNAs. Firstly, host miRNAs could suppress or facilitate viral replication [5, 6, 38]. Secondly, many DNA viruses, including herpesviruses, adenovirus, polyomaviruses and papillomavirus, have evolved to encode viral miRNAs to potentially control various phases of the viral life cycle, such as latency, reactivation, replication, etc. [42–46]. We do not expect VV would encode its viral miRNAs, similar to those typical cellular miRNAs, because this virus is replicated in the cytoplasm. Thirdly, both our present study and Dr. Grinberg’s report [20] showed that a suppression of host miRNA expression was followed by the reduction of Dicer protein during VV infection. Other DNA viruses may not suppress Dicer protein since this would affect the processing of their own viral miRNAs. Therefore, it is not surprising that VV, different from the other DNA viruses, uses a novel way to interact with miRNAs.

RNAi could serve as an innate antiviral mechanism in plants, fungi and animals. Human viruses, like plant viruses, encode suppressor proteins or RNAs that block or modulate the RNAi pathway [17]. Several mammalian viruses contain viral proteins with RSS activity, that usually involved two mechanisms: Dicer binding and siRNA binding [16]. The results of this study indicated that I7 protease of VV could cleave Dicer protein to facilitate Dicer degradation. Thus, VV I7 protease possesses RSS activity with a novel mechanism.

Suppression of VV replication slightly was demonstrated by exogenous expression of Dicer protein (Fig. 6A and 6B). However, knockdown of Dicer protein did not facilitate VV replication significantly (Fig. 6C and 6D). This may be simply due to the efficient cleavage of Dicer protein during VV infection (Figs. 1 and 2).

In conclusion, results in this study indicate that, during VV infection, the cleavage of Dicer protein by I7 protease facilitates Dicer degradation, and in turn, suppresses the processing of miRNAs.

## Supporting Information

S1 File This file contains Figures SI and SII. (DOCX)
