# Supplementary material for: Cleavage of Dicer Protein by I7 Protease during Vaccinia Virus Infection
Source: PLoS One. 2015 Mar 27;10(3):e0120390. doi: 10.1371/journal.pone.0120390 (PMC4376780; doi:10.1371/journal.pone.0120390)
Supplement: S1 File — (DOCX) [file pone.0120390.s001.docx]

**Supporting Information**

For File S1


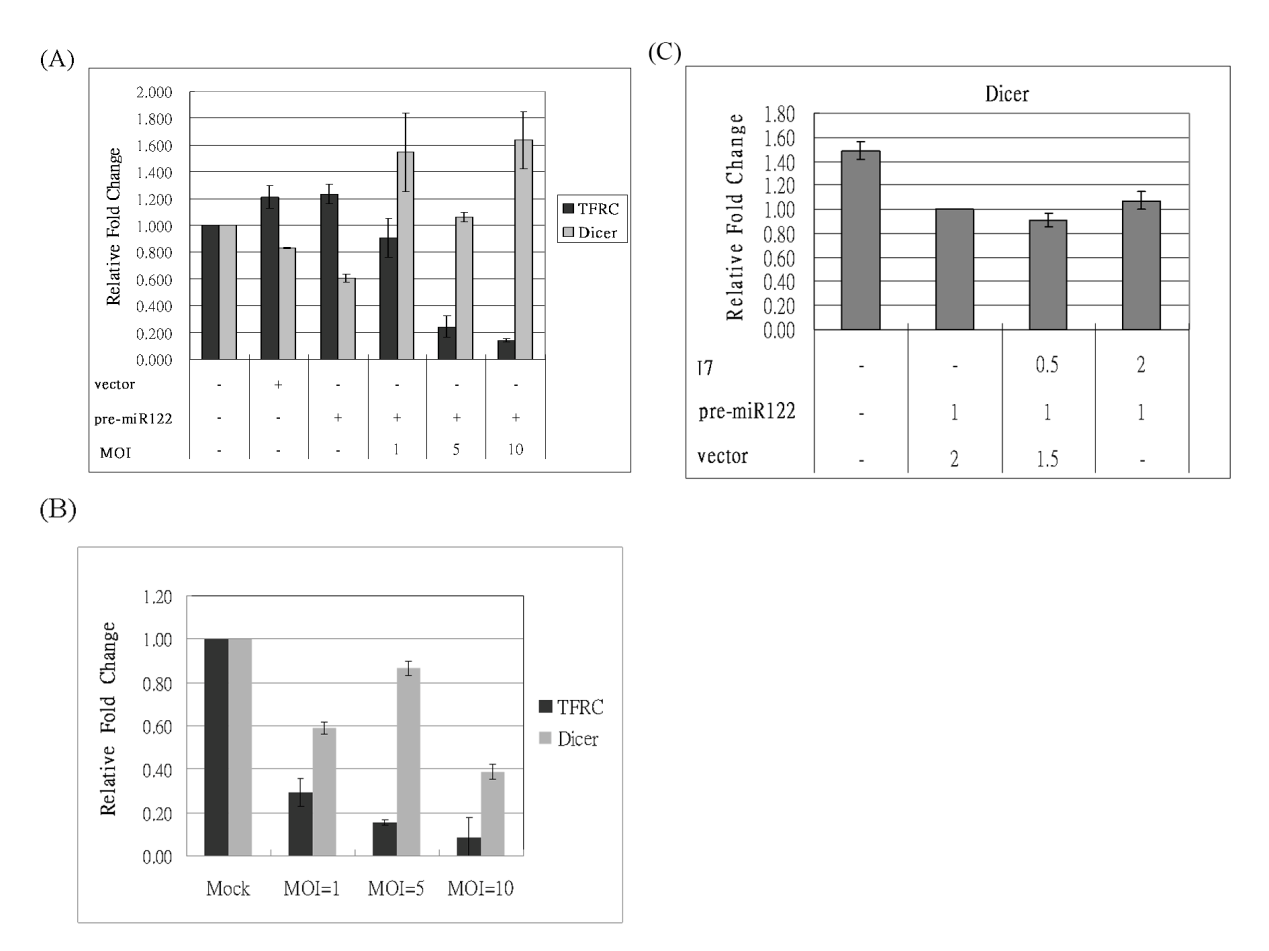


Fig. S1. **Unlike the TFRC mRNA decreased after VV infection dose-dependently, the Dicer mRNA was not specifically suppressed during VV infection or by I7 protease expression.** (A) HeLa cells were mock-transfected or transfected with 2 ug of empty vector or the plasmid expressing pre-miR122. Twenty-four hrs after transfection, the cells with pre-miR122 were mock infected or infected with VV in different dosage (M.O.I.=1, 5, 10). Twenty-four hrs after infection, mRNAs were extracted and converted into cDNA. Then, real-time PCR assay was performed to detect the mRNA amounts of TFRC or Dicer. (B) HuH7 cells were mock-infected or infected with VV in different dosage (M.O.I.=1, 5, 10). Twenty-four hrs after infection, mRNAs were extracted and converted into cDNA. Then, real-time PCR assay was performed to detect the mRNA amounts of TFRC or Dicer. (C) HeLa cells were mock-transfected or co-transfected with the plasmids expressing pre-miR122 and I7 protease with the indicated amount. Forty-eight hrs after transfection, mRNAs were extracted and converted into cDNA. Then, real-time PCR assay was performed to detect the mRNA amounts of TFRC or Dicer.


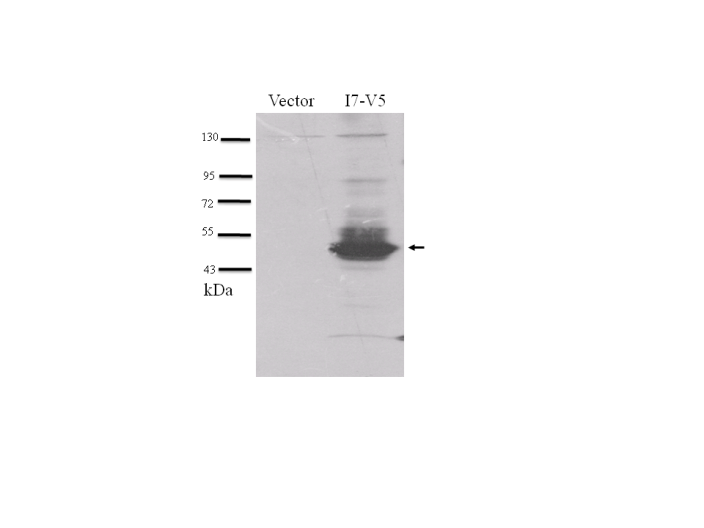


Fig. S2. **Expression of I7 protease.** HeLa cells were infected with recombinant vaccinia virus expressing T7 RNA polymerase and transfected with empty vector or the plasmid expressing I7 protease with a V5 tag at its C-terminus. Sixteen hrs after transfection, cell lysates were analyzed by SDS-PAGE and Western blotting with antibodies against the V5 tag.
